# Diabetes and the Risk of Multi-System Aging Phenotypes: A Systematic Review and Meta-Analysis

**DOI:** 10.1371/journal.pone.0004144

**Published:** 2009-01-07

**Authors:** Feng-Ping Lu, Kun-Pei Lin, Hsu-Ko Kuo

**Affiliations:** 1 Department of Geriatrics and Gerontology, National Taiwan University Hospital, Taipei, Taiwan; 2 Institute of Health Policy and Management, College of Public Health, National Taiwan University, Taipei, Taiwan; 3 Institute of Epidemiology, College of Public Health, National Taiwan University, Taipei, Taiwan; 4 Department of Internal Medicine, National Taiwan University Hospital, Taipei, Taiwan; 5 Division of Gerontology Research, National Health Research Institutes, Taipei, Taiwan; National Institute of Child Health and Human Development/National Institutes of Health, United States of America

## Abstract

**Background:**

Observational studies suggested an association between diabetes and the risk of various geriatric conditions (i.e., cognitive impairment, dementia, depression, mobility impairment, disability, falls, and urinary incontinence). However, the magnitude and impact of diabetes on older adults have not been reviewed.

**Methodology/Principal Findings:**

MEDLINE and PSYCINFO databases were searched through November 2007 for published studies, supplemented by manual searches of bibliographies of key articles. Population-based, prospective cohort studies that reported risk of geriatric outcomes in relation to diabetes status at baseline were selected. Two authors independently extracted the data, including study population and follow-up duration, ascertainment of diabetes status at baseline, outcomes of interest and their ascertainment, adjusted covariates, measures of association, and brief results. Fifteen studies examined the association of DM with cognitive dysfunction. DM was associated with a faster decline in cognitive function among older adults. The pooled adjusted risk ratio (RR) for all dementia when persons with DM were compared to those without was 1.47 (95% CI, 1.25 to 1.73). Summary RRs for Alzheimer's disease and vascular dementia comparing persons with DM to those without were 1.39 (CI, 1.16 to 1.66) and 2.38 (CI, 1.79 to 3.18), respectively. Four of 5 studies found significant association of DM with faster mobility decline and incident disability. Two studies examined the association of diabetes with falls in older women. Both found statistically significant associations. Insulin users had higher RR for recurrent falls. One study for urinary incontinence in older women found statistically significant associations. Two studies for depression did not suggest that DM was an independent predictor of incident depression.

**Conclusions/Significance:**

Current evidence supports that DM is associated with increased risk for selected geriatric conditions. Clinicians should increase their awareness and provide appropriate care. Future research is required to elucidate the underlying pathological pathway.

## Introduction

The prevalence and morbidities associated with DM continue to increase among older adults, which impose a significant burden on the health care system [1]–[3]. In addition to traditional vascular complications, growing epidemiologic evidence has suggested that DM could be independently associated with various aging phenotypes, or so-called “geriatric syndromes”, namely cognitive impairment [4]–[6], dementia [7], [8], depression [9], [10], mobility impairment or disability [11]–[14], falls [15], and urinary incontinence [16], [17]. These problems are highly prevalent in older people, especially frail older adults. They have detrimental effects on the quality of life, functional outcomes, and even mortality of the elderly [18]–[20].

However, the temporal relationship between DM and geriatric syndromes remained unanswered. A number of primary studies assessing the association between diabetes and separate geriatric outcomes had methodological flaws, such as a cross-sectional design [13], [21], [22], problematic diabetic ascertainment [23], [24], or inadequate consideration of potential confounders [25]. In addition, many studies enrolled nursing home residents [26] or clinical trial volunteers [27], thus having serious concerns of generalizability. A few systematic reviews have focused on the effect of diabetes on individual geriatric outcomes [28], [29], but they also presented several weaknesses, such as inclusion of cross-sectional studies or those without proper adjustment for major confounders.

We therefore conducted a systematic review and meta-analyses of prospective population-based studies examining the association between diabetes and the incidence of various geriatric conditions to verify the impact of DM on community-dwelling older adults.

## Methods

### Searching

We searched MEDLINE (1950 to November 2007) and PSYCINFO (1967 to November 2007) databases using combination of the following terms, both as key words and mapped to MeSH terms with explosion when possible: “cognitive impairment”, “cognition”, “cognition disorders”, “dementia”, “depression”, “depressive symptoms”, “depressive disorder”, “walking”, “musculoskeletal equilibrium”, “mobility limitation”, “physical function”, “activities of daily living”, “disability evaluation”, “disabled persons”, “functional decline”, “accidental falls”, and “urinary incontinence”. To identify prospective studies, we used the following index terms: “cohort studies”, “risk”, “group”, or “incidence”. Then the searches were combined with “diabetes mellitus” and “aged”. Additional references were found by reviewing bibliographies from identified articles. We considered articles published in any language.

### Selection

Individual study had to meet the following inclusion criteria: (1) population-based studies of community-dwelling older adults (i.e. persons aged 60 and over); (2) examination of the prospective effect of DM on individual geriatric syndrome; (3) DM being ascertained by combination of medical history, use of anti-diabetic medications, or laboratory tests (fasting glucose and/or glucose tolerance test). Studies with DM ascertainment based on self-reported history were excluded. To facilitate generalizability, we excluded studies conducted in specific population including hospital-based patients, institutionalized older adults, or pharmaceutical clinical trial volunteers. Mild cognitive impairment (MCI) was not included in cognitive outcomes due to lack of consistent diagnostic criteria. Studies without appropriate consideration or adjustment for potential confounders, especially cardiovascular confounders, were excluded.

### Validity Assessment and Data Abstraction

Two of the authors independently examined all identified articles. Following the Meta-analysis Of Observational Studies in Epidemiology (MOOSE) statement [30], we used a standardized reporting form to abstract the following data and quality indicators from each eligible study: first author and citation, country, study design, study population and follow-up duration, ascertainment of diabetes status at baseline, outcomes of interest and their ascertainment, adjusted covariates, measures of association, and brief results.

### Quantitative Data Synthesis

Selected papers were grouped and examined based on individual geriatric condition. A meta-analysis of studies was done if the results were presented in the same, combinable format. Data management and analysis were done using STATA version 9.0 software (Stata, College Station, Texas) and Review Manager 4.2 (The Cochrane Collaboration, Oxford, United Kingdom). We used fixed-effects models and DerSimonian and Laird random-effects models to calculate the pooled estimate across the studies. The χ-squared test was used to check for heterogeneity between the studies. Meta-regression was performed to assess the effect of several clinical factors on risk of geriatric outcomes. The possibility of publication bias was assessed using Begg's funnel plot and Egger's test. A careful qualitative appraisal was presented if the results between studies could not be appropriately combined.

## Results

### Flow of included studies

**Figure 1:**
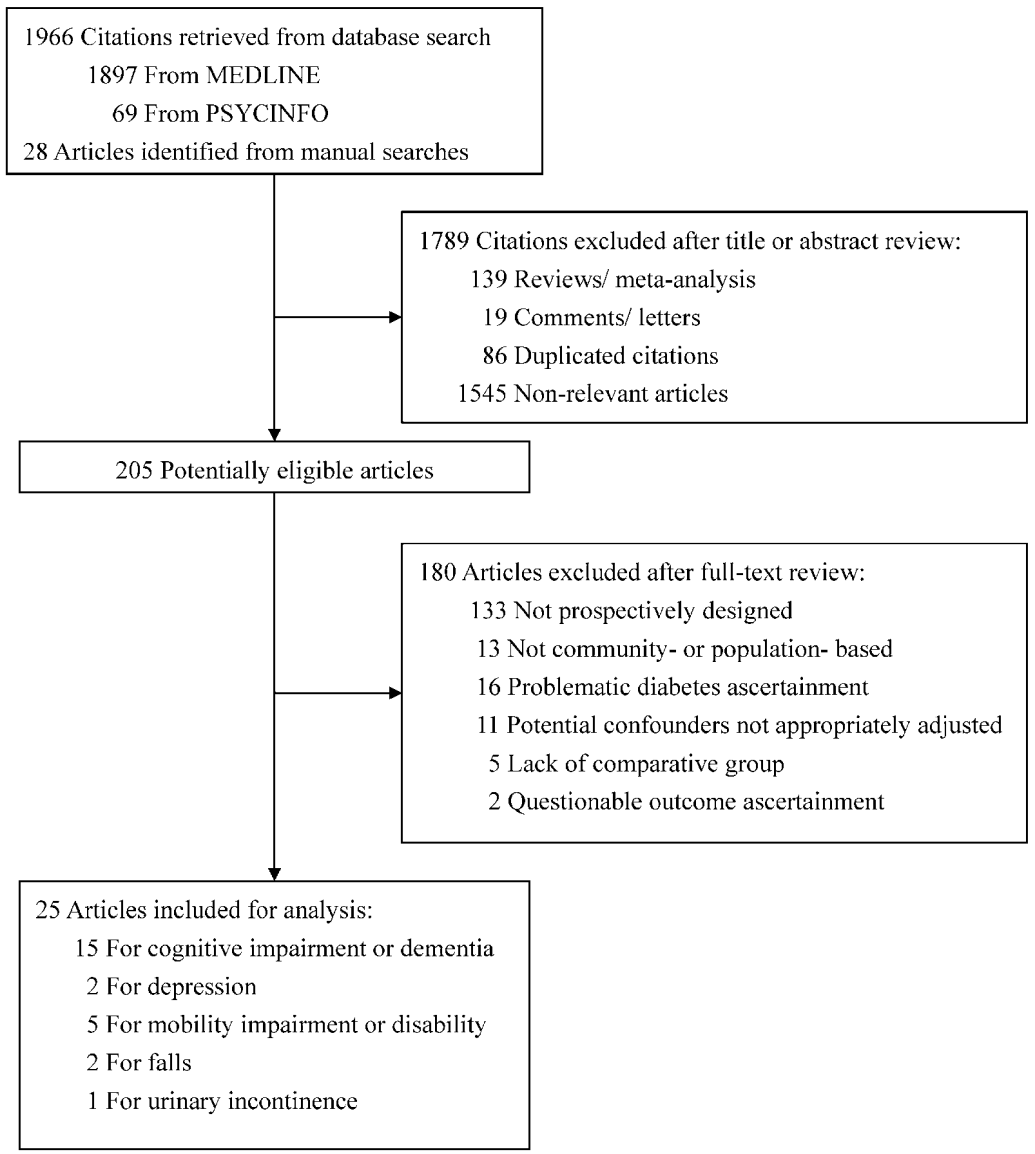
Flow diagram of selection process.

Based on the database search strategies, 1994 citations were retrieved. We identified 205 potentially relevant studies. Among these, 25 studies met the predefined selection criteria. Figure 1 showed the search and selection process. There were 15 studies included for an association between DM and cognitive dysfunction, two for depression, five for mobility impairment or disability, two for falls, and one for urinary incontinence, respectively.

### Cognitive Dysfunction

#### Changes in global cognition or individual cognitive domain

Seven prospective studies that assessed the association of DM and changes in cognitive performance (Table 1) have suggested a causal role of DM in the development of cognitive impairment [6], [31]–[36]. Measures of cognitive function in these studies included global cognition, such as Mini-Mental State Examination (MMSE) and the Telephone Interview for Cognitive Status (TICS), as well as individual cognitive domains, such as tests of memory, visuospatial ability, and frontal executive functions. Patients with DM were reported to have a faster decline in global cognitive function, as reflected by the scores of MMSE or TICS, after 2–7 years of follow-up [33], [35], [36].

**Table 1:** Population-based prospective studies for the association between diabetes and changes in cognitive performance.

Source	Study population	Mean age at baseline, yr	Follow-up, yr	Ascertainment of diabetes	Ascertainment of cognitive function	Covariates	Results (diabetic individuals had faster decline in …)
Haan et al, 1999 [31]	5888 elders from the Cardiovascular Health Study (CHS) (USA)	>65	5–7	Hx, Rec, FG/OGTT	Modified MMSE, DSST	Age, sex, race, incident stroke, education	DSST
Gregg et al, 2000 [6]	9679 community-dwelling elderly white women from the Study of Osteoporotic Fractures (SOF) (USA)	72	3–6	Hx, Mx	Modified MMSE, visuospatial/motor speed of processing (Trailmaking B, DSST)	Age, education, depression, visual impairment, stroke, baseline score	Visuospatial/ motor speed of processing (Trails Making B and DSST)
Fontbonne et al, 2001 [32]	961 healthy community-dwelling elders from the Epidemiology of Vascular Aging (EVA) Study (France)	65	4	Hx, Mx, FG	MMSE, visual attention (Trailmaking B), immediate verbal memory (Auditory Verbal Learning Test), visuospatial processing (Test of Facial Recognition), speed of processing (DSST), psychomotor speed (Finger Trapping Test), immediate visual memory (Benton Visual Retention Test), logical reasoning (Raven's Progressive Matrixes), and auditory attention (Paced Auditory Serial Addition Test)	Age, sex, education, baseline score, systolic blood pressure, BMI	Finger Trapping Test
Wu et al, 2003 [33]	1789 older Mexican Americans from the population-based Sacramento Area Latino Study on Aging (SALSA) project (USA)	71	2	Hx, Mx, FG	Modified MMSE, and verbal memory (Delayed Word-List Recall Test)	Age, sex, education, baseline cognitive score, CES-D score, acculturation, hypertension	Modified MMSE score and word-list test*
Kanaya et al, 2004 [34]	999 community-dwelling white adults enrolled in the Rancho Bernardo Study (USA)	70.7	4	Hx, Mx, FG/OGTT	MMSE, Verbal Fluency Test, and Trailmaking B	Age, education, BDI score, presence of ApoE epsilon 4 allele, baseline cognitive test score, current estrogen use for women	Verbal Fluency Test (association in women only)
Hassing et al, 2004 [35]	258 elderly individuals from the Origins of Variance in the Old-Old study (OCTO-Twin Study) (Sweden)	83	6	Rec, Mx	MMSE	Age, sex, education, smoking, angina, myocardial infarction, CHF, stroke, TIA	MMSE score
Logroscino et al, 2004 [36]	18999 elderly women from the Nurses' Health Study (USA)	74	2	Hx, Mx	TICS, test of verbal fluency (Animal Naming Test), delayed recall of a 10 word list, digit span backwards, and immediate/delayed recall of the East Boston memory test	Age, education, high cholesterol and hypertension, vitamin E supplement, age at menopause, BMI, smoking, antidepressant use, alcohol intake, post-menopausal hormone use, mental health index, energy-fatigue index, use of aspirin, NSAIDs	TICS

* Logistic regression model revealed that baseline DM was a predictor of major cognitive decline, defined as a 9-point or greater decrease in scores on Modified MMSE, 4-point or greater decrease in scores on the word-list test.

BDI indicates Beck Depression Inventory, BMI body mass index, CDR Clinical Dementia Rating, CES-D Center for Epidemiologic Studies Depression Scale, CHF chronic heart failure, CNS central nervous system, DSST Digit Symbol Substitution test, FG fasting glucose test, Hx self-report history of diabetes or a physician's diagnosis of diabetes, MMSE Mini-Mental Status Examination, Mx use of anti-diabetes medications including insulin, NFG non-fasting glucose test, OGTT oral glucose tolerance test, Rec diabetes mellitus ascertained from medical records, TIA transient ischemic attack, TICS Telephone Interview for Cognitive Status, WAIS Wechsler Adult Intelligence Scale.

However, tests of global cognitive function may not be sensitive enough to detect impairment in individual cognitive domains. Frontal executive functions, measured by such tests as Trailmaking B, Digit Symbol Substitution Test (DSST), and the word-list generation (verbal fluency), were selectively impaired by increased load of cardiovascular risk [37], [38]. Prospective population-based studies in our review consistently reported that baseline DM was associated with a faster decline in measures of executive function [6], [31], [32], [34].

Two studies reported increased risk of cognitive decline with the duration of diabetes [6], [36]. One of the two studies examining the association of insulin use and risk of cognitive decline suggested a positive association [6]. One study found that the association between diabetes and cognitive decline was attenuated by elevated levels of glycohemoglobin [34].

In short, DM was associated with decline in cognitive, especially executive, function. Although included studies had considered the impact of cardiovascular covariates on the association between DM and cognitive decline, not all studies had controlled for depressive symptoms or the use of psychotropic agents. Future studies should consider potential confounders and investigate the role of glycemic control of cognitive function.

#### All dementia, Alzheimer's disease (AD), and vascular dementia (VaD)

Eight original articles fulfilled the predefined criteria (Table 2) [8], [39]–[45]. All were prospective population-based studies conducted in Canada, United States, or Europe, with various follow-up periods ranging from 2.1 years to more than 10 years. Participants were older community-dwelling adults with mean baseline age varied from 69 to 83 years.

**Table 2:** Population-based prospective studies of diabetes as a risk factor for dementia in older adults.

Source	Study population	Mean age at baseline, yr	Follow-up, yr	Ascertainment of diabetes	Ascertainment of dementia	Covariates	All dementia, RR (95% CI)	Alzheimer's disease, RR (95% CI)	Vascular dementia, RR (95% CI)
Ott et al, 1999 [39]	6370 elderly persons from the community-based Rotterdam Study (Netherlands), 11% with DM	68.9	2.1	Mx, NFG, OGTT	DSM-III (dementia); NINCDS-ADRDA (AD); NINDS-AIREN (VaD)	Age, sex	1.9 (1.3–2.8) *	1.9 (1.2–3.1) *	2.0 (0.7–5.6) *
Luchsinger et al, 2001 [8]	1262 healthy Medicare beneficiaries residing in northern Manhattan (USA), 20% with DM	75.6	4.3	Hx , Mx	DSM-IV (dementia); NINCDS-ADRDA (AD); clinical judgment for stroke-associated dementia (VaD)	Gender, race, education, smoking, hypertension, heart disease, LDL level	NP	1.3 (0.84–1.88)	3.4 (1.70–6.91)
Hassing et al, 2002 [40]	702 elderly individuals from the population-based Origins of Variance in the Old-Old study (OCTO-Twin Study) (Sweden), 15% with DM	83	6–8	Rec, Mx	DSM-III-R (dementia); NINCDS-ADRDA (AD); NINDS-AIREN (VaD)	Age, sex, education, smoking, myocardial infarction, angina, CHF, hypertension, hypotension, TIA, stroke	NP	0.85 (0.36–2.02)†	3.63 (1.35–9.76)†
MacKnight et al, 2002 [41]	5574 elderly participants from the Canadian Study of Health and Aging (Canada), 9% with DM	74	5	Hx , Mx, Rec	DSM-III-R (dementia); NINCDS-ADRDA (AD); ICD-10 (VaD)	Age, sex, education, stroke, hypertension, and heart disease	1.26 (0.90–1.76)	1.30 (0.83–2.03)	2.03 (1.15–3.57)
Peila et al, 2002 [42]	2574 Japanese-American elderly men from the fourth exam cohort (1991–1993) of the Honolulu-Asia Aging Study (USA), 35% with DM	77	3	Hx, Mx, FG, OGTT	DSM-III-R (dementia); NINCDS-ADRDA (AD); CADDTC (VaD)	Age, education, ApoE epsilon 4 status, diabetes medications, alcohol/smoking status, midlife systolic blood pressure, cholesterol, BMI, ABI, stroke, CHD	1.5 (1.01–2.2)	1.8 (1.1–2.9)	2.3 (1.1–5.0)
Xu et al, 2004 [43]	1301 community elderly dwellers from the Kungsholmen project (Sweden), 8.8% with DM	81	4.7	Rec, Mx, NFG	DSM-III-R (dementia); NINCDS-ADRDA (AD); NINDS-AIREN (VaD)	Age, sex, education, stroke, heart disease, BMI, SBP, DBP, anti- hypertensive medications	1.5 (1.0–2.1)	1.3 (0.9–2.1)	2.6 (1.2–6.1)
Akomolafe et al, 2006 [44]	2210 community-dwelling dementia- free elders from Framingham Study Original cohort (USA), 9.1% with DM	70	12.7	Mx, NFG	DSM-IV (dementia); NINCDS-ADRDA (AD); CADDTC (VaD)	Age, sex, education, plasma homocysteine, SBP, BMI, current smoking, alcohol use, stroke, CVD	1.20 (0.74–1.96)	1.15 (0.65–2.05)	0.81 (0.18–3.70)
Hayden et al, 2006 [45]	3264 aged 65 or older adults from the community-based cohort of Cache County Study of Memory Health and Aging (CCSMHA) (USA), 10.5% with DM	74	3.2	Hx, Mx	DSM-III-R (dementia); NINCDS-ADRDA (AD); NINDS-AIREN (VaD)	Age, sex, education, ApoE epsilon 4 status, hypertension, high cholesterol, obesity, stroke, myocardial infarction, CABG	1.56 (0.90–2.56)	1.33 (0.66–2.46)	2.23 (0.88–5.17)‡

* Additional adjustments for education, BMI, alcohol/smoking status, hypertension, ABI, heart disease, stroke did not result in substantial changes of the estimates.

† Exclude 81 persons diagnosed with dementia at baseline.

‡ Diabetes increased the risk of VaD in females after adjustments (aHR 3.33, 95% CI 1.03–9.78) but not males.

ABI indicates ankle-to-brachial index, AD Alzheimer's disease, BMI body mass index, CADDTC California Alzheimer's Disease Diagnostic and Treatment Centers, CHD coronary heart disease, CHF chronic heart failure, DBP diastolic blood pressure, DM diabetes mellitus, FG fasting glucose test, Hx self-report history of diabetes or a physician's diagnosis of diabetes, LDL low density lipoprotein cholesterol, Mx use of anti-diabetes medications including insulin, NFG non-fasting glucose test, NINCDS-ADRDA National Institute of Neurological and Communicative Diseases and Stroke/Alzheimer's Disease and Related Disorders Association, NINDS-AIREN National Institute of Neurological Disorders and Stroke-Association Internationale pour la Recherche et l'Enseignement en Neurosciences, NP not performed, OGTT oral glucose tolerance test, Rec diabetes mellitus ascertained from medical records, RR risk ratio, SBP systolic blood pressure, TIA transient ischemic attack, VaD vascular dementia.

**Figure 2:**
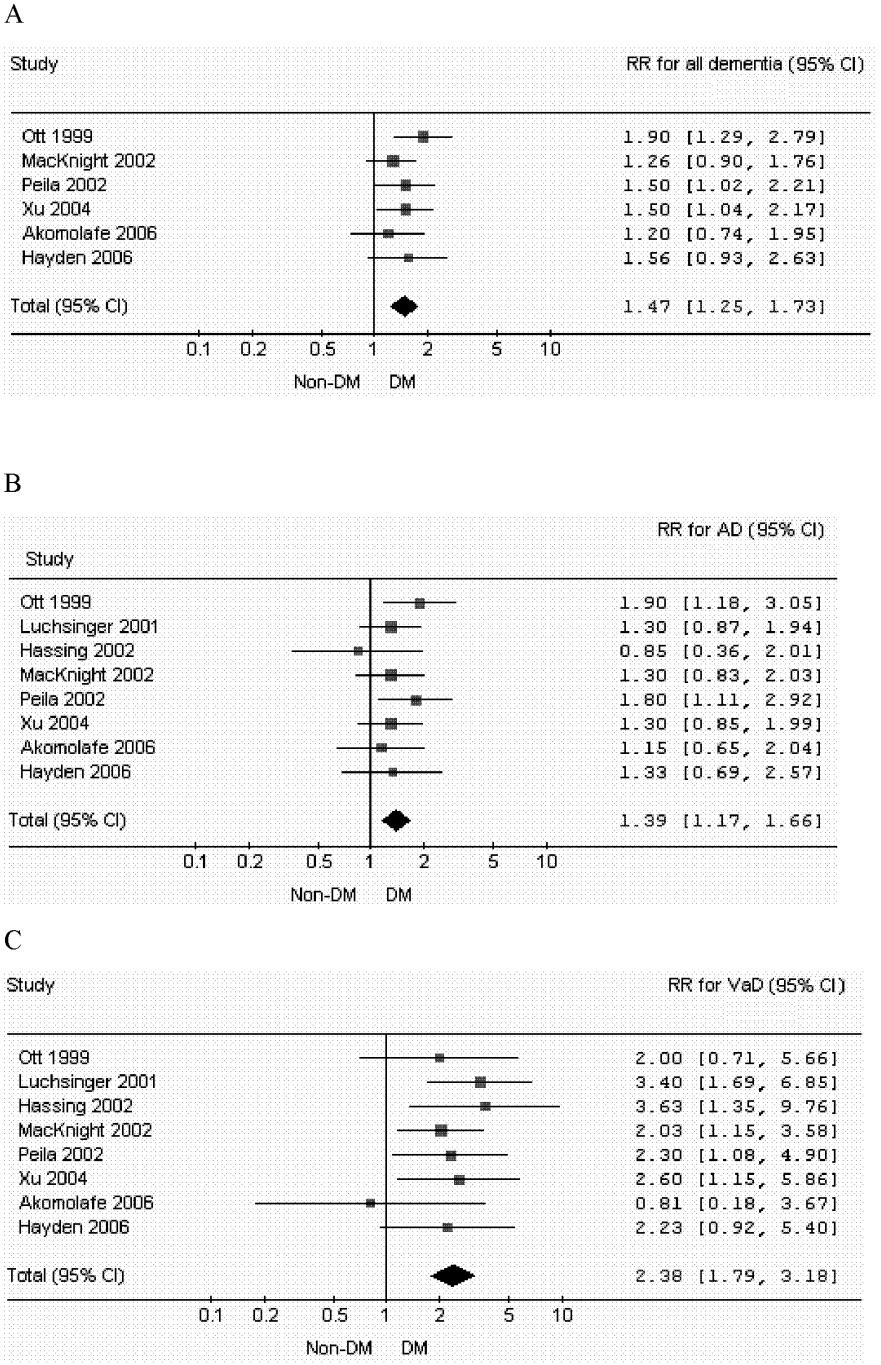
Adjusted relative risk for all dementia (A), Alzheimer's disease (B), and vascular dementia (C) in older diabetic adults compared with non-diabetics in prospective population-based studies.

Two studies [8], [40] did not provide risk estimate for all dementia. Results from the remaining six studies showed that DM was associated with a higher risk for all dementia, yet not all of them achieved statistical significance. The six studies used the same format, namely, risk ratios (RRs), or hazard ratios (HRs) for the risk of developing dementia for diabetes compared with non-diabetes. Two studies using logistic regression for analysis [41], [42]. The adjusted odds ratios (ORs) were considered as RRs since the incidence of dementia were not common in the study population [46]. In order to quantitatively analyze the results from various studies and increase the statistical power, we performed a meta-analysis to combine the reported results. Meta-analysis of the six studies showed that, the overall RR for all dementia when persons with DM were compared to those without was 1.47 (95% CI, 1.25 to 1.73) (Figure 2A). Test for heterogeneity using a χ-squared test showed a Q-test statistic of 3.269 with five degrees of freedom (p = 0.659), indicating that there was little evidence for heterogeneity among studies.

As for incident AD, two of the eight eligible studies reported diabetic patients had significantly higher risk of AD comparing to those without DM [39], [42]. Five studies reported increased, yet not achieving statistical significance, risk of AD comparing persons with DM to those without [8], [41]–[43], [45]. One study from Sweden reported decreased risk, though not significant, for AD among older diabetics [40]. It may be explained by many competing risks for AD among the oldest cohort (mean age 83 years). On the other hand, data reporting the association between DM and risk of VaD were more consistent. Six studies reported that DM was significantly associated with an increased risk of VaD [8], [40]–[43], [45]. The other two negative findings had small statistical power because relatively few cases of incident VaD were identified [39], [44]. Meta-analysis of the eight studies showed that the overall RRs for AD and VaD comparing persons with DM to those without were 1.39 (CI, 1.16 to 1.66, p<0.001) and 2.38 (CI, 1.79 to 3.18, p<0.001), respectively (Figure 2B, 2C). Test for heterogeneity did not reveal significant heterogeneity among studies.

A fixed effects model was selected for further meta-regression because prior test for between-study heterogeneity was not statistically significant. The meta-regressions showed no difference in effects between either country (non-USA versus USA), age, sex ratio, and study year on the risk of all dementia, AD, and VaD. For tests of publication bias, visual inspection of the Begg's funnel plots for all dementia, AD, and VaD did not reveal asymmetry (P>0.05). The Egger's tests were not statistically significant.

None of the eight studies examined the association between duration of diabetes and the risk of dementia. Five of the eight studies had considered the association between diabetes medications and risk of dementia [39], [41]–[44]. The results were heterogeneous. Insulin use was related to higher risk of dementia in two studies [39], [41]. One study found no effect of treatment on the risk of incident dementia [44]. Another study reported increased risk of dementia was associated with use of oral anti-diabetic medications [43].

In summary, we found that DM was associated with a 47% increased risk for all dementia, 39% for Alzheimer's dementia, and more than 2-fold risk for vascular dementia, among community-dwelling older adults. The association of DM to dementia was independent of cardiovascular comorbidities.

### Depression

Two eligible prospective studies are heterogeneous in terms of depression ascertainment (table 3) [47], [48]. Therefore, meta-analytic technique is not applicable. The longitudinal Zaragoza Dementia and Depression (ZARADEMP) project in Spain followed 4,803 community-dwelling older adults for 5 years [47]. Major depressive disorder was diagnosed by psychiatric diagnostic interview at 2- and 5-year follow-up. After controlling for age and sex, patients with DM had an increased risk (OR, 1.42 [CI, 1.04 to 1.93]) of incident depressive disorder. However, the associations attenuated after further adjustment (OR, 1.28 [CI, 0.91 to 1.79]). Investigators from the Health ABC Study followed 3,024 community-dwelling older adults for a mean of 5.9 years [48]. They found that, after controlling for basic demographics and life styles, DM was associated with a 30% increased risk (OR, 1.31 [CI, 1.07 to 1.61]) of incident depressed mood. Further adjustment for cardiovascular covariates attenuated the association (OR, 1.21 [CI, 0.98 to 1.49]). They found that the level of glycohemoglobin was independently associated with increased risk of recurrent depressed mood, but duration of DM and DM treatment did not have effect on the risk of recurrent depressed mood [48].

**Table 3:** Prospective studies of diabetes as a risk factor for depression in community-dwelling older adults.

Source	Study population	Mean age at baseline, yr	Follow-up, yr	Ascertainment of diabetes	Ascertainment of depression	Covariates	Results, OR (95% CI)
De Jonge et al, 2006 [47]	4803 community-dwelling elders from the ZARADEMP project (Spain)	73.5	5	Hx, Mx	MDD by psychiatric diagnostic interview at 2- and 5-yr follow-up	Age, sex, partner, education, hypertension, smoking, statin use, and cognitive functioning	1.28 (0.91–1.79)
Maraldi et al, 2007 [48]	2522 community-dwelling subjects from the Health, Aging, and Body Composition (Health ABC) study (USA)	73.6	5.9	Hx, Mx, FG/OGTT	Antidepressant use at follow-up or CESD-10> = 10	Age, sex, race, study site, baseline CES-D score, hypertension, cerebrovascular disease, ankle-brachial index, obesity, cystatin-C level, IL-6 levels, 6-m walking speed, and cognitive functioning	1.21 (0.98–1.49)

CESD-10 indicates a 10-item subset of the standard Centre for Epidemiologic Studies Depression Scale, FG fasting glucose test, Hx self-report history of diabetes or a physician's diagnosis of diabetes, IL-6 interleukin-6, MDD major depressive disorder, Mx anti-diabetes medications including insulin, OGTT oral glucose tolerance test, Rec diabetes mellitus ascertained from medical records, RR risk ratio.

Although both studies suggested that DM patients had an increased risk for depression, the association diminished after additional consideration of cardiovascular confounders, suggesting that baseline DM was not independently associated with incident depressive disorder. Depression is known for a chronic, relapsing disorder in older adults with diabetes [49]. The two studies did not consider depressive episodes between each follow-up interval, nor did they provide information regarding depressive episodes prior to study entry. The effect of DM on incident depression was difficult to determine based on current literatures.

To explain the association between DM and depression observed in cross-sectional studies, a reverse causal relationship has been proposed. The notion that depression was an independent risk factor for DM, rather than a consequence of having diabetes, was supported by a few prospective studies and meta-analysis over the past decade [50]–[54]. Future prospective studies with proper design to investigate the temporal relation between DM and depression are mandated.

### Mobility Impairment and Disability

We identified five studies that fulfilled the predefined criteria (Table 4) [11], [55]–[58]. Because the physical outcome measures and format of results were heterogeneous, meta-analysis was not performed.

**Table 4:** Prospective studies for the association between diabetes and changes in mobility or physical function in older adults.

Source	Study population	Mean age at baseline, yr	Follow-up, yr	Ascertainment of diabetes	Ascertainment of physical function	Covariates	Results, RR (95% CI) (or diabetic individuals had faster decline in …)
Volpato et al, 2002 [11]	729 physically impaired community-dwelling older women from the Women's Health and Aging Study (WHAS) (USA)	77	3	Hx, Mx, Rec, A1c	ADLs, self-reported ability to perform mobility task (walking 1/4 mile, climbing steps); physical performance measures (usual 4-m walking speed, 5 chair stands, balance test)	Age, race, smoking, BMI, depressive symptoms, cognitive impairment, knee OA, hip fracture, baseline performance score, HTN, stroke, CAD, CHF, PVD, peripheral nerve dysfunction, visual impairment	Mobility disability: 1.63 (1.12–2.36)*; ADL disability: 2.18 (1.33–3.6)†; summary physical performance score
Gregg et al, 2002 [56]	8344 white elder women from the Study of Osteoporotic Fractures (SOF) (USA)	71.4	12	Hx, Mx	Self-reported ability to perform functional tasks (walking 1/4 mile, climbing 10 steps, household chores, shopping, and cooking meals)	Age, marital status, education, BMI, baseline physical functioning, physical activity level, estrogen use, visual impairment, poor cognitive function, CAD, stroke, depression, arthritis	Inability to perform any task: 1.42 (1.23–1.65)
Wu et al, 2003 [57]	1789 older Mexican Americans from the Sacramento Area Latino Study on Aging (SALSA) (USA)	70	2	Hx, Mx, FG	ADLs, IADLs	Age, sex, household income, BMI, waist-to-hip ratio, CES-D score, HTN, stroke	ADLs, IADLs
Forrest et al, 2006 [55]	5178 white elder women from the Study of Osteoporotic Fractures (SOF) (USA)	70.1	10	Hx, Mx	Physical performance measures (usual 6-m walking speed, 5 chair stands)	Age, weight, height, height loss, SBP, smoking, baseline performance, arthritis, ever use of thyroid supplement or estrogen	Walking speed, time to complete 5 chair stands
Figaro et al, 2006 [58]	2895 well-functioning older adults from the Health, Aging and Body Composition (Health ABC) study (USA)	73.6	3.5	Hx, Mx, FG/OGTT	Self-reported ability to climbing 10 steps or walking 1/4 mile	Age, sex, race, BMI, baseline performance score, smoking, use of anti-inflammatory drugs, CHF, PVD, CAD, current estrogen use, statin use	Not significant

* 336 women with severe mobility disability at baseline were excluded from the analysis.

† 170 women with severe ADL disability at baseline were excluded from the analysis.

A1c indicates glycosylated hemoglobin, ABI ankle-brachial index, BMI body mass index, CAD coronary artery disease, CES-D Center for Epidemiologic Studies Depression Scale, CHF chronic heart failure, FG fasting glucose test, HTN hypertension, Hx self-report history of diabetes or a physician's diagnosis of diabetes, MMSE Mini-Mental Status Examination, Mx anti-diabetes medications including insulin, NFG non-fasting glucose test, OGTT oral glucose tolerance test, Rec diabetes mellitus ascertained from medical records, PVD peripheral vascular disease, SBP systolic blood pressure.

Most of the studies suggested that DM was associated with higher risk of mobility impairment and physical disability among older adults. The two studies reporting changes in objective physical performance measures were limited to older women [11], [55]. Result from the prospective Women's Health and Aging Study (WHAS) suggested that, during a 3-year period, women with diabetes had significant greater decline (38% per year) in objective physical performance, including walking speed, chair stands, and balance test, than non-diabetics [11]. Forrest et al. analyzed data from the Study of Osteoporotic Fractures (SOF) and found that, over 10 years, diabetes patients had greater decline in walking speed and ability to complete 5 chair stands, compared to their non-diabetic counterparts [55].

In addition to objectively measured physical performance, DM was associated with decline in self-reported functional outcomes. Gregg and colleagues followed 8,344 older women in the SOF cohort for more than 10 years, and suggested that patients with DM had higher risk of inability to perform one or more major functional tasks (walking 1/4 mile, climbing 10 steps, performing household chores, shopping, and cooking meals) [56]. Data from the WHAS showed that DM was associated with an increased risk of mobility disability (RR, 1.63 [CI, 1.12 to 2.36]) and activities of daily living (ADL) disability (RR, 2.18 [CI, 1.33 to 3.6]) over 3 years [11]. Another longitudinal study, the Sacramento Area Latino Study on Aging (SALSA) that followed 1,789 older Mexican Americans for 2 years, also found that diabetic patients had higher annual rate of decline in ADL and instrumental activities of daily living (IADL) [57]. On the contrary, investigators from the Health ABC study followed 2,895 high-functioning older adults for 3.5 years and found that DM was not associated with incident mobility disability [58]. This negative result may be explained, at least in part, by the enrollment of well-functioning older adults at baseline and a relatively short follow-up period (3.5 years).

Three studies examined the association between duration of diabetes and risk of functional decline [11], [56]–[57]; two of them found significant associations [11], [57]. One study reported that insulin use was not significantly associated with a higher risk of incident functional decline [56]. One study found that the risk of incident disability associated with diabetes was significantly attenuated after adjustment for levels of glycohemoglobin [11].

While most major covariates had been considered in these studies, the influence of diabetic duration and severity were not consistently reported. In addition, several medical conditions (e.g. obstructive lung disease) and disease severity that may affect patient's mobility were not considered. In sum, most studies suggested that diabetes was independently associated with functional decline, including performance-based physical function and self-reported functional disability, among older adults. Future studies should collect comprehensive data, utilize standardized measurement, and recruit men and women that are more representative of the older population to facilitate generalizability.

### Falls

There were two studies that fulfilled the eligibility criteria (table 5) [59], [60]. Both suggested that DM was associated with an increased risk of falls and recurrent falls, independent of established fall risk factors. Moreover, both studies found that insulin use was a major risk factor for falls.

**Table 5:** Prospective studies of diabetes as a risk factor for recurrent falls in community-dwelling older adults.

Source	Study population	Mean age at baseline, yr	Follow-up, yr	Ascertainment of diabetes	Ascertainment of falls	Covariates	Results, RR (95% CI)
Schwartz et al, 2002 [59]	9249 women from the Study of Osteoporotic Fractures (SOF) (USA)	74	7.2	Hx, Mx	Falls monitored every 4 months by postcard or telephone	Age, tandem walk score, tandem stand, loss of pressure sensitivity, CAD, stroke, arthritis, history of fainting, grip strength, positive GDS, near depth perception, sedatives/ anxiolytics use	Non-insulin-treated diabetes: 1.18 (0.87–1.60); insulin-treated diabetes: 2.76 (1.52–5.01)
Volpato et al, 2005 [60]	878 community -dwelling, disabled women from the Women's Health and Aging Study (WHAS) (USA)	78	3	Hx, Mx, Rec, A1c	Self report of falls at 6 semi-annual interviews	Age, race, education, smoking, overweight, obesity, hypertension, use of anti-hypertensives, stroke, PAD, peripheral nerve dysfunction, knee osteoarthritis pain categories, visual impairment, MMSE score, fall in 12 months prior to baseline interview, ADL disability, physical performance score, knee strength	Diabetes: 1.69 (1.18–2.43); non-insulin-treated diabetes: 1.34 (0.87–2.1); insulin-treated diabetes: 2.73 (1.61–4.63)

A1c indicates glycosylated hemoglobin, ADL activity of daily living, BMI body mass index, CAD coronary artery disease, GDS Geriatric Depression Score, Hx self-report history of diabetes or a physician's diagnosis of diabetes, MMSE Mini-Mental Status Examination, Mx anti-diabetes medications including insulin, PAD peripheral arterial disease, Rec diabetes mellitus ascertained from medical records, RR risk ratio.

Schwartz et al. [59] followed 9,249 women enrolled in the SOF for 7 years. Incident falls were ascertained every 4 months by postcard. They found that DM was associated with an increased risk of falling. After controlled for physical performance, chronic conditions, cardiovascular covariates, and use of sedatives or anxiolytics, the association between non-insulin-treated diabetes and falls was significantly attenuated (RR, 1.18 [CI, 0.87 to 1.60]). The risk of falls comparing insulin-treated diabetic patients versus controls remained essentially unchanged (RR, 2.76 [CI, 1.52 to 5.01]). Another prospective WHAS, recruiting 1,002 disabled community-dwelling women aged 65 years and over, assessed incident falls semiannually for 3 years [60]. After adjustment for traditional risk factors and diabetes complications, women with diabetes had a higher probability of any fall (RR, 1.38 [CI, 1.04 to 1.81) and of recurrent falls (RR, 1.69 [CI, 1.18 to 2.43]), compared to women without diabetes. Risk of recurrent falls was particularly higher among insulin-treated older women (RR, 2.73 [CI, 1.61 to 4.63]). The pooled adjusted RR for recurrent falls was 2.74 (CI, 1.85 to 4.07) when older women with insulin-treated diabetes were compared to controls.

Although both studies suggested the relation of DM to falls, some confounders (e.g., psychotropics use, cognition) were not consistently adjusted. In addition, both studies had weakness in terms of generalizability because the study population was confined to older women. Future studies should address on a broader sampling strategy and enroll older men as well.

### Urinary Incontinence (UI)

One prospective study was identified based on our search strategy (table 6). Lifford et al. [61] analyzed 81,845 women from the NHS cohort who had reported information on urinary function 4 years apart. The adjusted relative risk of incident UI was significantly greater (RR, 1.21 [CI, 1.02 to 1.43]) in women with DM than those without. The risk of developing very severe UI was even more substantial in women with diabetes (RR, 1.97 [CI, 1.24 to 3.12]). They also found that the risk of incontinence increased with the duration of DM.

**Table 6:** Prospective studies of diabetes as a risk factor for urinary incontinence in community-dwelling older adults.

Source	Study population	Mean age at baseline, yr	Follow-up, yr	Ascertainment of diabetes	Ascertainment of urinary incontinence	Covariates	Results, RR (95% CI)
Lifford et al, 2005 [61]	53650 female nurses from Nurses' Health Study (NHS) cohort (USA)	62	4	Hx, Mx	Any UI: urine leakage at least weekly; severe UI: at least weekly leakage of a quantity of urine enough to wet the underwear; very severe UI: at least weekly leakage of a quantity of urine enough to wet outer clothing or the floor	Age, BMI, race, functional status, stroke, waist-to-hip ratio, hysterectomy, parity, smoking, hormone use	Any UI: 1.21 (1.02–1.43); severe UI: 1.40 (1.15–1.71); very severe UI: 1.97 (1.24–3.12)

BMI indicates body mass index, Hx self-report history of diabetes or a physician's diagnosis of diabetes, Mx anti-diabetes medications including insulin, RR risk ratio, UI urinary incontinence.

The result suggested that DM was associated with higher risk of UI in older women. However, types of UI (stress, urge, or overflow) were not reported. Therefore it was difficult to identify the pathophysiologic linkage between DM and incontinence. In addition, the investigators did not take into account several covariates, such as cognitive function and mood status, which might interfere with the development of UI among older adults. Future well-designed prospective studies, with standardized definition and measurement for voiding function, are warranted to elucidate the relationship between DM and UI.

## Discussion

We performed a systematic review of prospective population-based studies addressing the association between DM and various geriatric conditions among community-dwelling adults. The risk for cognitive impairment, dementia, mobility decline, or disability increased with diabetes compared to those without. The pooled risk for all dementia, Alzheimer's disease, and vascular dementia were 1.4 to 2.4 times higher in older diabetic adults compared to non-diabetics. Identified studies reporting the association between DM and the incidence of falls or urinary incontinence were limited to older women, and all indicate a positive association. Available evidence did not suggest that DM was an independent predictor of incident depression.

### Mechanisms

There is biological evidence supporting the causality that DM may lead to geriatric conditions. Several molecular mechanisms have been proposed to explain the mediation between DM and hyperglycemia-induced tissue damage, including the advanced glycation end products (AGEs) [62]. Chronic hyperglycemia may induce oxidative stress or through aforementioned mechanisms, thereby causing subsequent systematic endothelial dysfunction and diabetic vascular complications [62], [63]. DM-associated metabolic derangements, AGEs, systemic inflammation, along with traditional diabetic vascular complications, may play critical roles in the development of geriatric conditions.

Diabetes may exert its negative impact on cognitive function through several pathways. Hyperglycemia promotes the formation of AGEs, which were found in senile plaques and intracellular neurofibrillary tangles [64]. Increase or decrease in plasma glucose concentrations can affect cognitive function [65], [66]. Insulin resistance and subsequent hyperinsulinemia also contribute. Insulin inhibits the degradation of beta-amyloid (the main product of the AD process) through competitive inhibition of insulin-degrading enzyme in the brain, thus increasing amyloid protein deposition in plaques [67]. Hyperinsulinemia may also activate inflammatory network in the periphery and the brain, thereby increases the risk of AD [67]. DM-related vasculopathy in the brain may lead to lacunes, leukoariosis, large artery ischemic stroke, and cortical atrophy. These structural changes are known to be risk factors for cognitive dysfunction and dementia.

Diabetes may affect physical performance in older adults through several mechanisms. In animal studies, non-enzymatic glycation alters the structures and functions of myosin and actins, which further interfere with muscle contraction [69]–[72]. In addition, hyperglycemia- induced chronic inflammation status exerts negative impact on skeletal muscle function [73], [74]. These were in line with clinical observations that older diabetic adults have poorer muscle quality compared with their non-diabetic counterparts, and lose their muscle strength and quality more rapidly, especially those with longer disease duration and poorer glycemic control [75]. Furthermore, cerebral vasculopathy, peripheral artery disease, peripheral polyneuropathy, autonomic neuropathy, and retinopathy are also responsible for the mobility limitations and falls among older adults with diabetes.

The effect of DM on voiding function is more complex, which may involve pathophysiological changes in the detrusor muscle, urothelium, central and autonomic nervous systems, and blood supply to the bladder [76]. Hyperglycemia may cause an increased volume of urine, polyuria, or detrusor instability. Additionally, diabetes may contribute to the development of urinary incontinence through associated cognitive dysfunction, functional limitation, or medical comorbidities, even though the lower urinary tract is intact. Hence the impact of DM on urinary incontinence is likely multi-factorial.

### Strengths and Limitations

To our knowledge, the present review is the first one that summarizes prospective data relating DM to geriatric conditions. Cukierman and colleagues conducted a systematic review of prospective studies to examine the relationship between DM and changes in cognitive function [77]. They found that people with diabetes have a greater rate of cognitive decline and an increased risk of future dementia (RR, 1.6 [CI, 1.4 to 1.8]), which was concordant with our results. However, studies conducted in a historical cohort or with questionable baseline diabetes ascertainment were included in their analysis.

In this review, we included only prospectively-designed studies exploring the causal relationship of DM to various aging phenotypes. Therefore, the temporal relationship was appropriate (i.e. DM preceded the incidence of geriatric conditions). In addition, studies with questionable DM ascertainment were excluded in order to minimize misclassification of patients. Because there are also possible non-causal explanations for the association between DM and geriatric conditions, we excluded studies without appropriate adjustment for potential confounders, especially cardiovascular comorbidities. Hence the results provide more precise estimates of the impact of DM on older adults.

Our searches did not include conference papers and thesis, which may bring a selection bias. Geriatric syndromes are complex multi-factorial problems. Cognitive status, mobility, sensory function, and psychosocial status of older adults are all crucial to the development of geriatric conditions. Nevertheless, not all included studies have thoroughly considered these factors. In addition, studies included in our review did not consistently report disease-related factors, such as duration of DM, status of glycemic control, and treatment. These factors may confound the outcomes of interest. Finally, literatures regarding DM to several outcomes (e.g., falls and UI) were limited to older women, hence the generalizability was limited.

### Future Implications

The evidence supported that older diabetic adults have an increased risk for selected multi-system aging phenotypes, which were associated with substantial morbidity and adverse outcomes among older population [18]. In addition, some of the problems (e.g., cognitive dysfunction, mobility impairment) may significantly interfere with disease management [78], [79]. While physicians try to do their best to manage DM in older adults, this additional evidence may encourage increased awareness, management and patient education in their daily practice.

Future research should attempt to explore the underlying mechanisms linking DM to geriatric conditions, and address complex interaction between risk factors. DM as a shared risk factors across different geriatric conditions raises the possibility of shared pathophysiological mechanisms across aging phenotypes, such as AGEs formation, oxidative stress, systemic inflammation, and vasculopathy. On the other hand, test of causal relationship can only rely on interventional studies showing that intensive glycemic control can prevent or delay the incidence of geriatric conditions. However, given that older adults are more vulnerable to adverse effect of strict glucose control, modification of other cardiovascular factors may be an alternative. Prior investigation has confirmed the benefit of tight blood pressure control on cardiovascular disease risk reduction among patients with type 2 diabetes [80]. Angiotensin-converting-enzyme (ACE) inhibitors and statins are also promising in vasculoprotective effect among diabetic patients [81], [82]. More randomized control studies are needed to determine whether these therapies alone or in combination could reduce or delay the development of multi-system aging phenotypes.

### Conclusion

Despite methodological limitations of the observational studies reviewed, the consistency of reported relationship between DM and selected geriatric conditions among community-dwelling older adults across studies support the proclaim that DM is a major risk factor for multi-system aging phenotypes. Primary care physician should be aware and appropriately manage these problems. Future research is required to elucidate the underlying pathological pathway linking DM to geriatric conditions. Interventional studies with glucose-lowering therapy are needed to test the hypotheses that intensive glycemic control can reduce adverse geriatric outcomes.
